# Muscle and bone characteristics of a Chinese family with spinal muscular atrophy, lower extremity predominant 1 (SMALED1) caused by a novel missense *DYNC1H1* mutation

**DOI:** 10.1186/s12920-023-01472-4

**Published:** 2023-03-07

**Authors:** Yazhao Mei, Yunyi Jiang, Zhenlin Zhang, Hao Zhang

**Affiliations:** Shanghai Clinical Research Center of Bone Disease, Department of Osteoporosis and Bone Diseases, Shanghai Sixth People’s Hospital, Shanghai Jiao Tong University School of Medicine, 200233 Shanghai, China

**Keywords:** *DYNC1H1*, SMALED1, Whole-exome sequencing, Sanger sequencing, Bone metabolism

## Abstract

**Background:**

Spinal muscular atrophy, lower extremity predominant (SMALED) is a type of non-5q spinal muscular atrophy characterised by weakness and atrophy of lower limb muscles without sensory abnormalities. SMALED1 can be caused by dynein cytoplasmic 1 heavy chain 1 (*DYNC1H1*) gene variants. However, the phenotype and genotype of SMALED1 may overlap with those of other neuromuscular diseases, making it difficult to diagnose clinically. Additionally, bone metabolism and bone mineral density (BMD) in patients with SMALED1 have never been reported.

**Methods:**

We investigated a Chinese family in which 5 individuals from 3 generations had lower limb muscle atrophy and foot deformities. Clinical manifestations and biochemical and radiographic indices were analysed, and mutational analysis was performed by whole-exome sequencing (WES) and Sanger sequencing.

**Results:**

A novel mutation in exon 4 of the *DYNC1H1* gene (c.587T > C, p.Leu196Ser) was identified in the proband and his affected mother by WES. Sanger sequencing confirmed that the proband and 3 affected family members were carriers of this mutation. As leucine is a hydrophobic amino acid and serine is hydrophilic, the hydrophobic interaction resulting from mutation of amino acid residue 196 could influence the stability of the DYNC1H1 protein. Leg muscle magnetic resonance imaging of the proband revealed severe atrophy and fatty infiltration, and electromyographic recordings showed chronic neurogenic impairment of the lower extremities. Bone metabolism markers and BMD of the proband were all within normal ranges. None of the 4 patients had experienced fragility fractures.

**Conclusion:**

This study identified a novel *DYNC1H1* mutation and expands the spectrum of phenotypes and genotypes of *DYNC1H1*-related disorders. This is the first report of bone metabolism and BMD in patients with SMALED1.

## Introduction

Distal hereditary motor neuropathy (dHMN), also known as distal spinal muscular atrophy (SMA), is a phenotypically and genetically heterogeneous group of diseases characterised by degeneration of the motor component of peripheral nerves [1]. The most common phenotype of dHMN is slowly progressing distal limb muscle weakness and atrophy with minimal or no sensory involvement, usually accompanied by foot deformities [2]. To date, more than 30 genes associated with dHMN have been identified (https://neuromuscular.wustl.edu/index.html) [1]. These genes encode proteins that perform essential functions in motor neurons and axons such as axonal transport, RNA processing, and protein folding [3]. Spinal muscular atrophy, lower extremity predominant (SMALED), a type of dHMN, is a group of autosomal dominant non-5q SMA disorders characterised by lower limb weakness and wasting without sensory abnormalities. Several genes including dynein cytoplasmic 1 heavy chain 1 (*DYNC1H1*, OMIM 600,112), bicaudal D homolog 2 (*BICD2*, OMIM 609,797), and transient receptor potential cation channel subfamily V member 4 (*TRPV4*, OMIM 605,427) have been shown to be associated with SMALED [4–6].

Cytoplasmic dynein is a ~ 1.4 MDa complex consisting of a homodimer of dynein heavy chain (DHC) and several smaller non-catalytic subunits that is responsible for the retrograde transport of cargo in axons and dendrites and for converting the energy from ATP hydrolysis into movement [7]. As *DYNC1H1* has an essential role in cargo transport, it is related to neuronal development, morphology, and survival [8]. Since the first report of a mutation in *DYNC1H1* was described in 2010 in relation to autosomal dominant SMALED1 [9], numerous articles have reported *DYNC1H1* mutations in neurologic diseases [10, 11] such as axonal Charcot-Marie-Tooth disease type 2O (CMT2O, OMIM 614,228), malformation of cortical development (MCD, OMIM 614,563), and autosomal dominant mental retardation 13 (MRD13, OMIM 614,563). Because of the considerable variability and overlap in phenotypes, the clinical diagnosis of a *DYNC1H1*-related disorder can be challenging even for experienced neurologists and geneticists [11].

In this report, we describe a Chinese family in which 5 individuals from 3 generations had congenital muscle atrophy or even lameness without cognitive dysfunction or sensory impairments. By combining whole-exome sequencing (WES) with Sanger sequencing, we identified the causative mutation and confirmed the diagnosis of SMALED1.

## Materials and methods

### Subjects

This study was approved by the Ethics Committee of the Shanghai Sixth People’s Hospital Affiliated to Shanghai Jiao Tong University School of Medicine. Written informed consent was obtained from all adult participants and the children’s parents. All methods were performed in accordance with the tenets outlined in the Declaration of Helsinki.

**Fig. 1:**
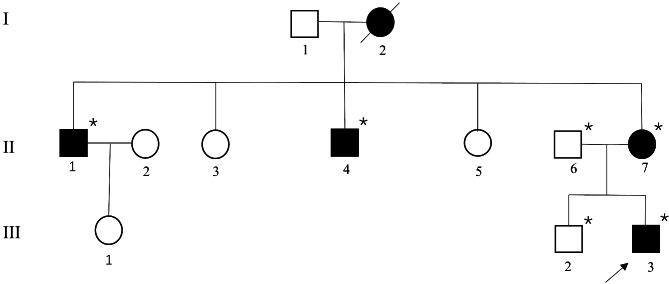
Pedigree of the family with SMALED1. Filled and open symbols represent affected and open symbols unaffected individuals, respectively; and squares and circles indicate males and females, respectively. The proband is indicated by an arrow. Slashes indicate deceased individuals. *DNA available

The 8-year-old male proband and 3 affected and 2 unaffected family members from a non-consanguineous Chinese pedigree (Fig. 1) were included in this study. Before participating in the study, written informed consent was obtained from the 6 family members. Six DNA samples were obtained from the peripheral blood of each subject with the QuickGene DNA whole blood kit (Kurabo Industries, Osaka, Japan) and a Nucleic Acid Isolation system (QuickGene-610 L; Autogen, Holliston, MA, USA). We performed physical examination of the patients and recorded detailed medical histories. Body height and weight were adjusted to sex- and age-specific Z-scores using reference data from the Chinese Han ethnic group [12]. Montreal Cognitive Assessment (MoCA) was administered to evaluate cognitive function.

### Laboratory and radiographic examination of the proband

Biochemical indices including complete blood count; levels of serum calcium (Ca), phosphonium (P), and alkaline phosphatase (ALP); liver and kidney functions; and levels of creatine kinase (CK) and its MB isoenzyme (CK-MB), 25-hydroxyvitamin D (25OHD), the intact parathyroid hormone (PTH) bone formation marker serum osteocalcin (OC), and the bone resorption marker serum beta cross-linked C-terminal telopeptide of type 1 collagen (β-CTX) were measured. Measurements of bone mineral density (BMD) of the lumbar spine and left femur neck were performed by dual-energy X-ray absorptiometry (Lunar iDXA, GE Healthcare, Madison, WI, USA). The results were transformed into age- and sex-specific Z-scores using reference data [13]. X-ray radiographs of thoracic and lumbar vertebrae, hips, knees, and ankles were obtained. The left leg muscle was examined by magnetic resonance imaging (MRI). Electromyography was performed and nerve conduction was analysed.

### WES

WES was performed on genomic DNA isolated from the peripheral blood of the proband, his parents, and his unaffected brother to identify the pathogenic gene mutation. The SureSelectXT Reagent kit was used to hybridise the SureSelectXT Human All Exon V6 (both from Agilent Technologies, Santa Clara, CA, USA) with the DNA library for WES capture. Polymerase Chain Reaction (PCR) was used to amplify the exon DNA library. High-throughput sequencing was performed on the Hiseq/NovaSeq platform (Illumina, San Diego, CA, USA) according to the manufacturer’s instructions with a 2 × 150 bp sequencing protocol. Reads were mapped to the human reference genome (GRCH38/hg38). Variants were annotated with Annovar (https://annovar.openbioinformatics.org/en/latest/) [14] and allele frequency, pathogenicity, and protein function were evaluated.

### Sanger sequencing

Sanger sequencing and PCR were used to confirm the putative mutation in *DYNC1H1* identified by WES. A primer pair (forward, 5′-TGATGGGATCTCTTTGGAGACCA-3′ and reverse, 5′-TTTGGCTTTTCTCCACGCTCAT-3′) designed with Web-based Primer 3 software (https://bioinfo.ut.ee/primer3-0.4.0/) was used for PCR amplification. Direct sequencing was performed on the proband, his parents, and his unaffected brother using BigDye Terminator Cycle Sequencing Ready Reaction Kit v. 3.1 (Applied Biosystems, Foster City, CA, USA) on an ABI 3730XL automated sequencer (Thermo Fisher Scientific, Waltham, MA, USA). Two affected uncles of the proband were screened for the identified mutation site. The Polyphred program was used to check for single nucleotide polymorphisms (SNPs). Amino acid conservation was analysed with the Universal Protein Resource (https://www.uniprot.org/). Polyphen-2 (http://provean.jcvi.org), SIFT (http://sift.jcvi.org), MutationTaster (https://www.mutationtaster.org/), and CADD (https://cadd.gs.washington.edu/snv) were used to predict the pathogenicity of the mutation. The novel mutation was verified using the ExAC program (http://exac.broadinstitute.org).

### Structural **analysis of DYNC1H1 protein**

The protein sequence of DYNC1H1 was obtained from the Uniprot database (https://www.uniprot.org/) as a FASTA file. Three-dimensional structure homology modelling and visualisation of the native and mutant proteins were performed using AlphaFold software (https://alphafold.ebi.ac.uk/).

## Results

### Clinical features

**Fig. 2:**
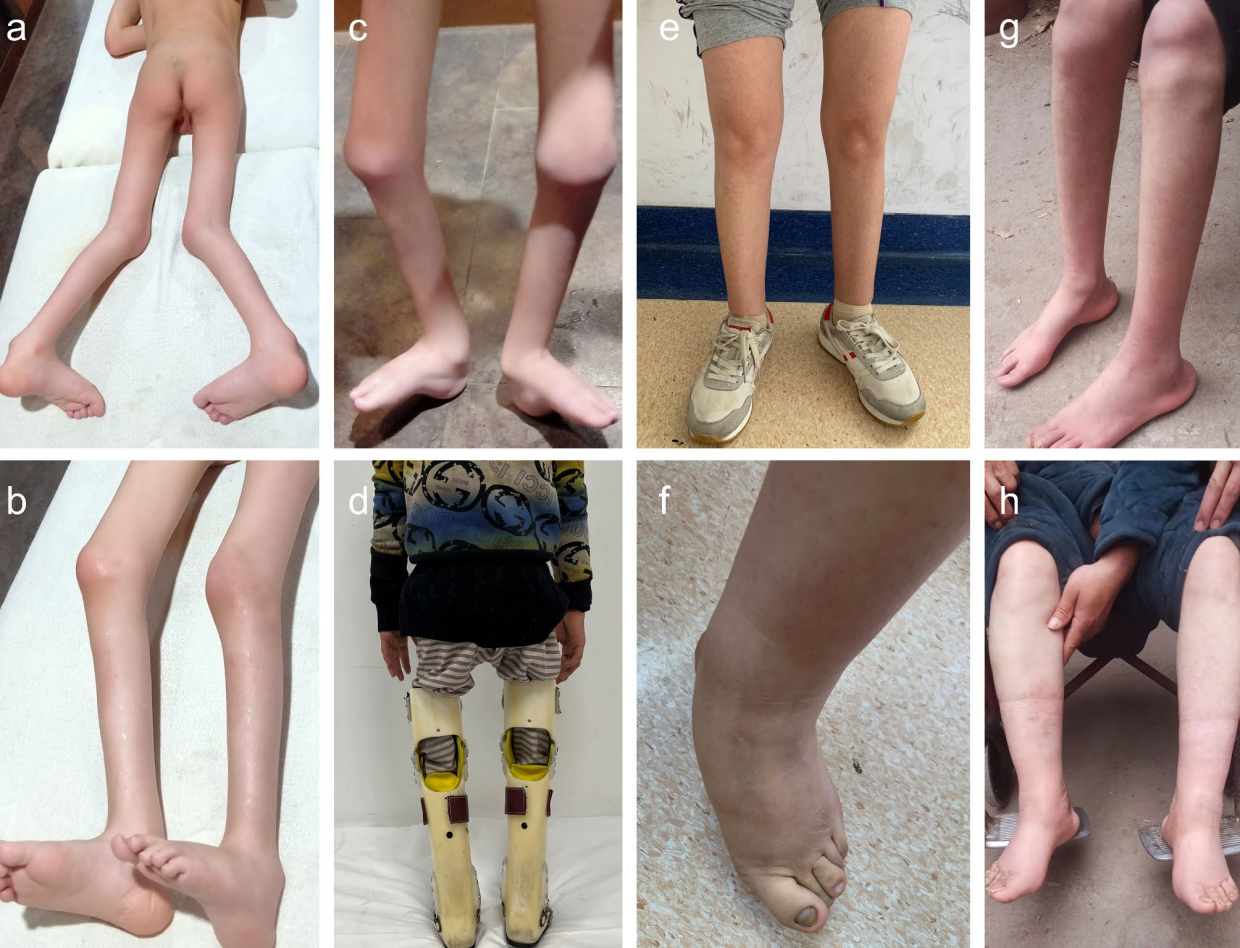
Photographs of the proband, his mother, and 2 uncles. (a–d) The proband had predominant lower limb atrophy, talipes calcaneovarus, and knee flexion contractures. He stood on the ground with his heels and could only walk with the assistance of ankle fixation braces. (e, f) The proband’s mother had milder lower limb atrophy and clubfeet. She was able to walk independently with a waddling gait starting from the age of 8 years old. (g, h) The proband’s 2 uncles had lower limb atrophy and clubfeet

The primary clinical characteristics of the proband and his affected family members are summarised in Table 1. The proband was born of non-consanguineous parents at 34 weeks of gestation, and his elder brother was normal. He had severe congenital talipes calcaneovarus at birth that improved slightly by the time he was 1 year old, likely as a result of myofascial massage. Hip and feet X-ray radiography and brain MRI were performed when he was 1 year old and were normal. However, he developed progressive weakness and atrophy of the lower extremity muscle with muscle tension in his feet. His motor development was severely delayed, and he was unable to walk or stand even with support. Before the proband came to our clinic at the age of 8 years, genome-wide copy number variation analysis and targeted next-generation sequencing had been performed when he was 2 and 3 years old, respectively, and no pathogenic variant related to the symptoms had been identified. A physical examination revealed clubfeet (talipes calcaneovarus), symmetrical muscle weakness, and atrophy primarily involving the lower limbs that made him unable to stand without ankle fixation braces. In addition, he had significant swelling of knee joints because he had moved by crawling for a long period of time (Fig. 2). His MoCA score was 27, which showed that his cognitive ability was unimpaired. Since birth he had not experienced any fractures. His height was 116 cm (Z-score: −2.20), weight was 17.7 kg (Z-score: −1.65), and head circumference was 52 cm. Muscle strength and tendon reflexes in the upper limbs were preserved but lower limb deep tendon reflexes were weakened. Other features such as fasciculations, sensory disturbances, ataxia, and consciousness disorder were not observed. The mother of the proband (II-7), who was 33 years old, had milder congenital clubfeet (pes cavus) and muscle atrophy in the lower extremities, which prevented her from walking independently until the age of 8 years. However, she still had mild congenital foot deformities (pes cavus) and a waddling gait as well as weakened lower limb deep tendon reflexes. Similarly, the proband’s 2 uncles (II-1 and II-4) had mild pes cavus and muscle atrophy in the lower extremities. Neither could walk independently until the age of 8 years. His mother and 2 uncles did not present other features such as fasciculations, neurologic deficits, sensory disturbances, ataxia, consciousness disorder, or fragility fractures. The deceased grandmother of the proband had had a similar phenotype.

**Table 1:** Primary clinical characteristics of the proband and his affected family members

	Proband	Mother	Uncle 1	Uncle 2	Reference
Sex	Male	Female	Male	Male	/
Age at the first visit (years)	8	33	42	34	/
Height (cm) (Z-score)	116 (− 2.20)	163	N.A.	168	/
Weight (kg) (Z-score)	17.7 (− 1.65)	40	N.A.	N.A.	/
Head circumference (cm)	52	52	N.A.	N.A.	/
Foot deformity	Talipes calcaneovarus	Pes cavus	Pes cavus	Pes cavus	/
Motor ability	Complete loss	Delayed	Delayed	Delayed	/
Gait	Unable to walk	Waddling gait	Waddling gait	Waddling gait	/
Muscle weakness and atrophy	Severe	Mild	Mild	Mild	/
Deep tendon reflexes	Weakened	Weakened	N.A.	N.A.	/
Sensory disturbances	No	No	No	No	/
Cognitive impairment	No	No	No	No	/
Fragility fractures	No	No	No	No	/
Creatine kinase (U/l)	91	N.A.	N.A.	N.A.	21–190
Creatine kinase isoenzymes (U/l)	22.3	N.A.	N.A.	N.A.	0.0–25.0
Calcium (mmol/l)	2.34	N.A.	N.A.	N.A.	2.25–2.75^a^
Phosphate (mmol/l)	1.53	N.A.	N.A.	N.A.	1.29–1.94^a^
ALP (U/l)	213	N.A.	N.A.	N.A.	116–380^a^
25OHD (ng/ml)	29.62	N.A.	N.A.	N.A.	≥ 0.0
PTH (pg/ml)	15	N.A.	N.A.	N.A.	15–65
OC (ng/ml)	96.49	N.A.	N.A.	N.A.	53.6–183.7^b^
β-CTX (ng/l)	2593.00	N.A.	N.A.	N.A.	1090–2940^b^
L1–L4 BMD (g/cm^2^) (Z-score)	0.654 (0.6)	N.A.	N.A.	N.A.	/
Left femur neck BMD (g/cm^2^) (Z-score)	0.754 (0.3)	N.A.	N.A.	N.A.	/

Abbreviations: 25OHD, 25-hydroxyvitamin D; ALP, alkaline phosphatase; β-CTX, beta cross-linked C-terminal telopeptide of type 1 collagen; BMD, bone mineral density; N.A., not available; OC, osteocalcin; PTH, parathyroid hormone

^a^Reference for children [39]

^b^Reference for boys between the ages of 8 and 9.9 years [40]

### Laboratory and radiographic examination of the proband

**Fig. 3:**
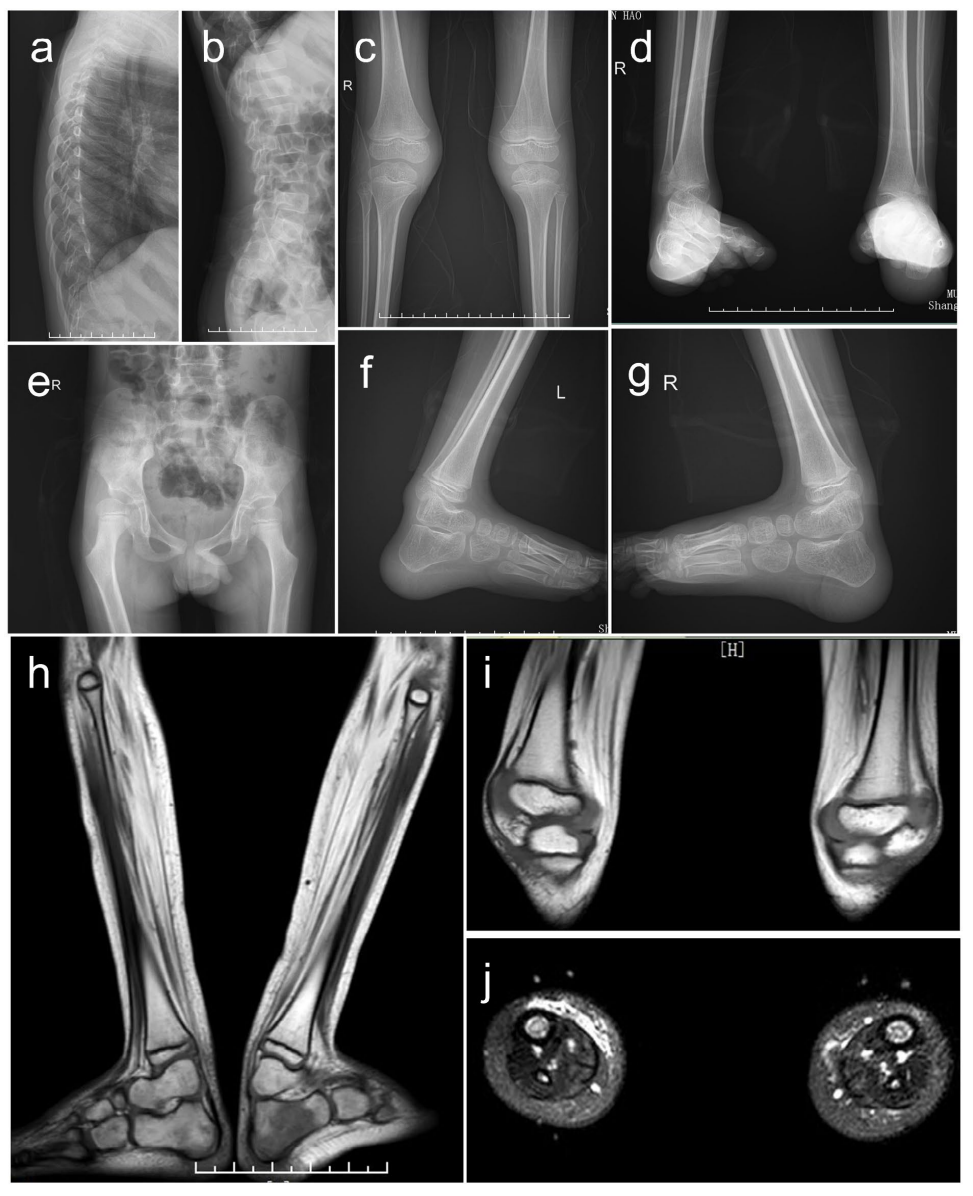
X-ray radiography and leg muscle MRI of the proband. (a, b, e) X-ray radiography of thoracic and lumbar vertebrae as well as hips was normal and showed no fractures. (c, d, f, g) X-ray radiography of the knees showed soft tissue swelling and ankles showed talipes calcaneovarus and poor alignment of the tibial talus joints. (h) Coronal T1W MRI of calf muscle with severe atrophy and fatty infiltration. Bone marrow oedema of the left calcaneus resulting from the ankle fixation braces that the proband had worn for a long time can be seen. (i) Coronal T1W MRI of distal thigh muscle with severe atrophy and fatty infiltration in posterior thigh muscles. (j) Axial T2W MRI of calves with severe atrophy and subcutaneous fat thickening

Clinical laboratory evaluation of the proband revealed average blood count, creatine kinase level, and liver and kidney functions (Table 1). His Z-scores for BMD were within the normal range (0.6 in the lumbar spine and 0.3 in the femur neck). X-ray radiography of thoracic and lumbar vertebrae as well as hips was normal and showed no fractures; meanwhile, knees showed soft tissue swelling and ankles showed talipes calcaneovarus and poor alignment of the tibial talus joints (Fig. 3). Left leg muscle MRI showed severe atrophy and fatty infiltration (Fig. 3). Sensory and motor nerve conduction studies revealed a decrease in compound muscle action potential amplitude of lower limb nerves. Electromyography (EMG) showed chronic neurogenic impairment of lower extremities. No significant abnormalities of sensory and motor nerve conduction were observed in upper limbs by EMG.

### WES data analysis

Variations including single-nucleotide variants (SNVs) and small insertions or deletions (indels) were identified using GATK HaplotypeCaller (https://gatk.broadinstitute.org/hc/en-us/articles/360037225632-HaplotypeCaller) and filtered according to the filtering scheme recommended by the software. We identified 133,853 SNVs in the target region, of which 15,899 were non-synonymous and 7689 were splice site variants. The family history suggested variants with an autosomal dominant mode of inheritance, and we identified 162 non-synonymous and splice site variants as candidate SNVs. Using protein function and pathogenic mutation prediction software, we ultimately identified a novel heterozygous missense mutation of the *DYNC1H1* gene (c.587T > C, p.Leu196Ser). The mutation was present in the proband and his affected mother, consistent with an autosomal dominant mode of inheritance.

### Validation of *DYNC1H1* germ line mutation

**Fig. 4:**
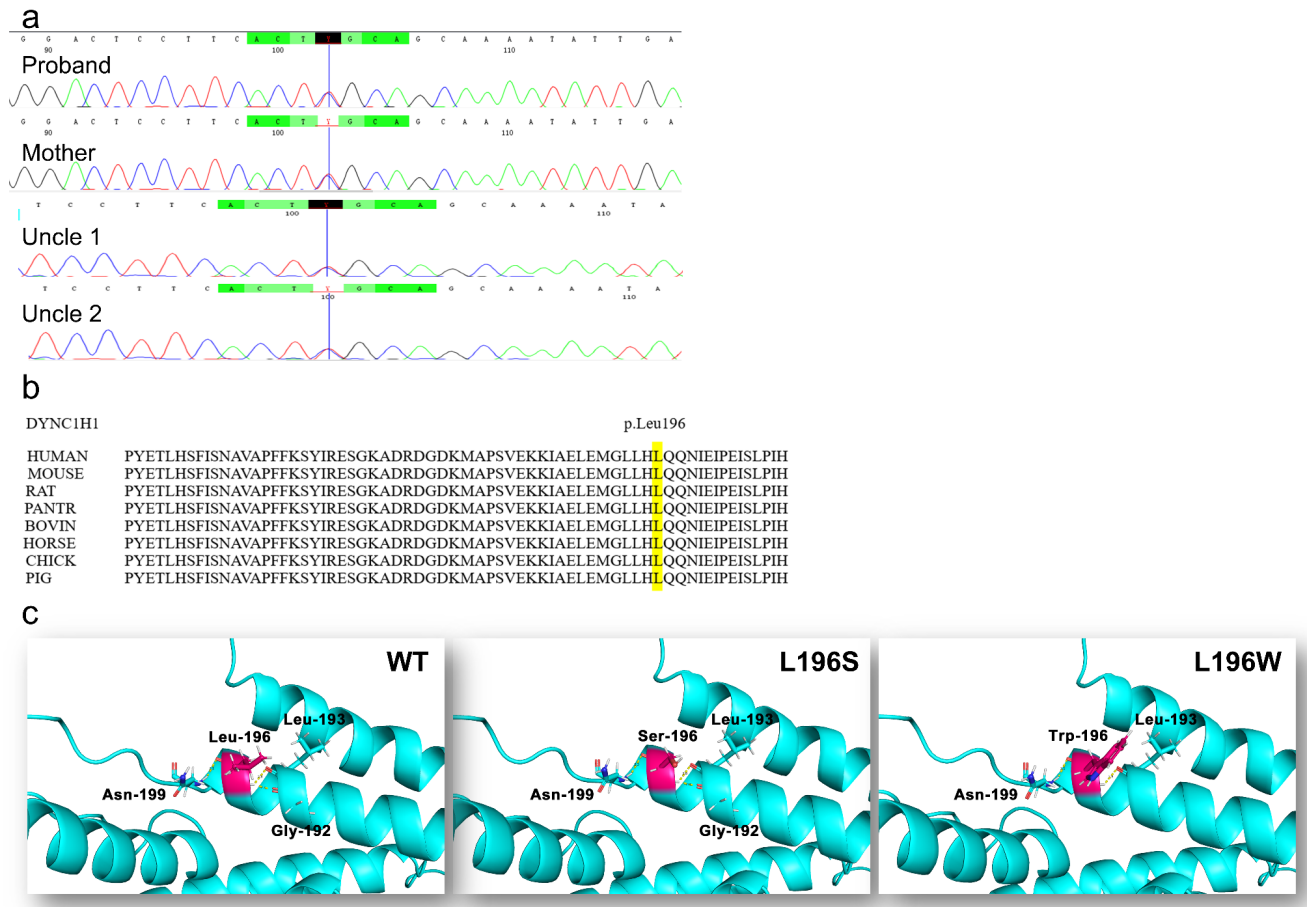
Sanger sequencing of the *DYNC1H1* variant. (a) Results of Sanger sequencing show the *DYNC1H1* mutation (c.587T > C, p.Leu196Ser) in the proband, his mother, and 2 uncles. (b) Amino acid sequence alignment of DYNC1H1 protein from different species reveals that the Leu196 residue is highly conserved. (c) Three-dimensional structure of the analogous region of the human cytoplasmic dynein N-terminal dimerisation domain. The yellow dotted lines represent the hydrogen bond

Sanger sequencing results of the 4 affected family members for the *DYNC1H1* gene were consistent with those obtained by WES. The heterozygous c.587T > C (p.Leu196Ser) mutation was detected in the proband and 3 relatives of the family (Fig. 4a). The variant is located in a highly conserved domain (Fig. 4b) and is predicted as “disease-causing,” by PolyPhen2, Mutation Taster, SIFT, and CADD (Table 2). American College of Medical Genetics and Genomics (ACMG) guidelines (15) classify the mutation (c.587T > C, p.Leu196Ser in *DYNC1H1*) as “likely pathogenic” as it belongs to PM2 (Absent from controls in Exome Sequencing Project, 1000 Genomes, and ExAC), PP1 (Co-segregation with the disease in multiple affected family members in a gene with a definitive role in a Mendelian disorder), PP2 (Missense variant in a gene that has a low rate of benign missense variation), PP3 (Multiple lines of computational evidence support a deleterious effect on the gene or gene product), and PP4 (Patient’s phenotype or family history is particular for a disease with a single genetic etiology).

**Table 2:** Prediction of the variant identified by WES and confirmed by Sanger sequencing

Gene	Transcript	Position	Variant	Function	dbSNP	ExAC database	SIFT	PolyPhen	Mutation Taster	CADD score
*DYNC1H1*	NM_001376	Chr:14, 101,979,787	L196S	Missense	Novel	Novel	Damaging	Probably damaging	Disease causing	29.3

### Structural analysis of DYNC1H1 protein

A model of the human cytoplasmic dynein N-terminal dimerisation domain is shown in Fig. 4c. As predicted by AlphaFold, the conversion of leucine to serine had no apparent effect on protein structure. Leucine is a hydrophobic whereas serine is a hydrophilic amino acid; consequently, hydrophobic interactions of the protein may be altered by the p.Leu196Ser mutation.

## Discussion

The proband in our study was sick at birth and underwent various examinations at multiple hospitals. However, a diagnosis was not confirmed until he came to our department at the age of 8 years along with his family members. Our initial diagnosis was dHMN based on his severe and predominant lower limb atrophy and foot deformities. WES, which enables detection of all known protein-coding genes in a single experiment, has facilitated the discovery of causal variants in rare diseases [16]. We used WES to identify the causative mutation in the proband, his parents, and his unaffected brother, and confirmed the results by Sanger sequencing. Based on the proband’s phenotype and relevant literature, we identified a novel mutation (c.587T > C, p.Leu196Ser) in the *DYNC1H1* gene that is predicted to be likely pathogenic according to ACMG guidelines. Based on these findings, we made a definitive diagnosis of SMALED1.

The primary clinical feature of SMALED is involvement of the motor nervous system in the lower extremities that results in atrophy and weakness of the lower extremity muscles starting from infancy. Patients usually have foot deformities and retardation in movement but retain motor ability in the face, neck, chest, back, abdominal muscles, and upper limb muscles; moreover, the sensory system is rarely or never involved and cognitive ability is unaffected. The 4 affected patients in our study had partially overlapping clinical manifestations. Muscle biopsy has limited value for diagnosing SMALED compared with leg muscle imaging as the results are often equivocal and could lead to a misdiagnosis of congenital myopathy [17]. The leg muscle MRI of the proband in our study showed severe atrophy and fatty infiltration, which contributed to the diagnosis of SMALED.

**Fig. 5:**
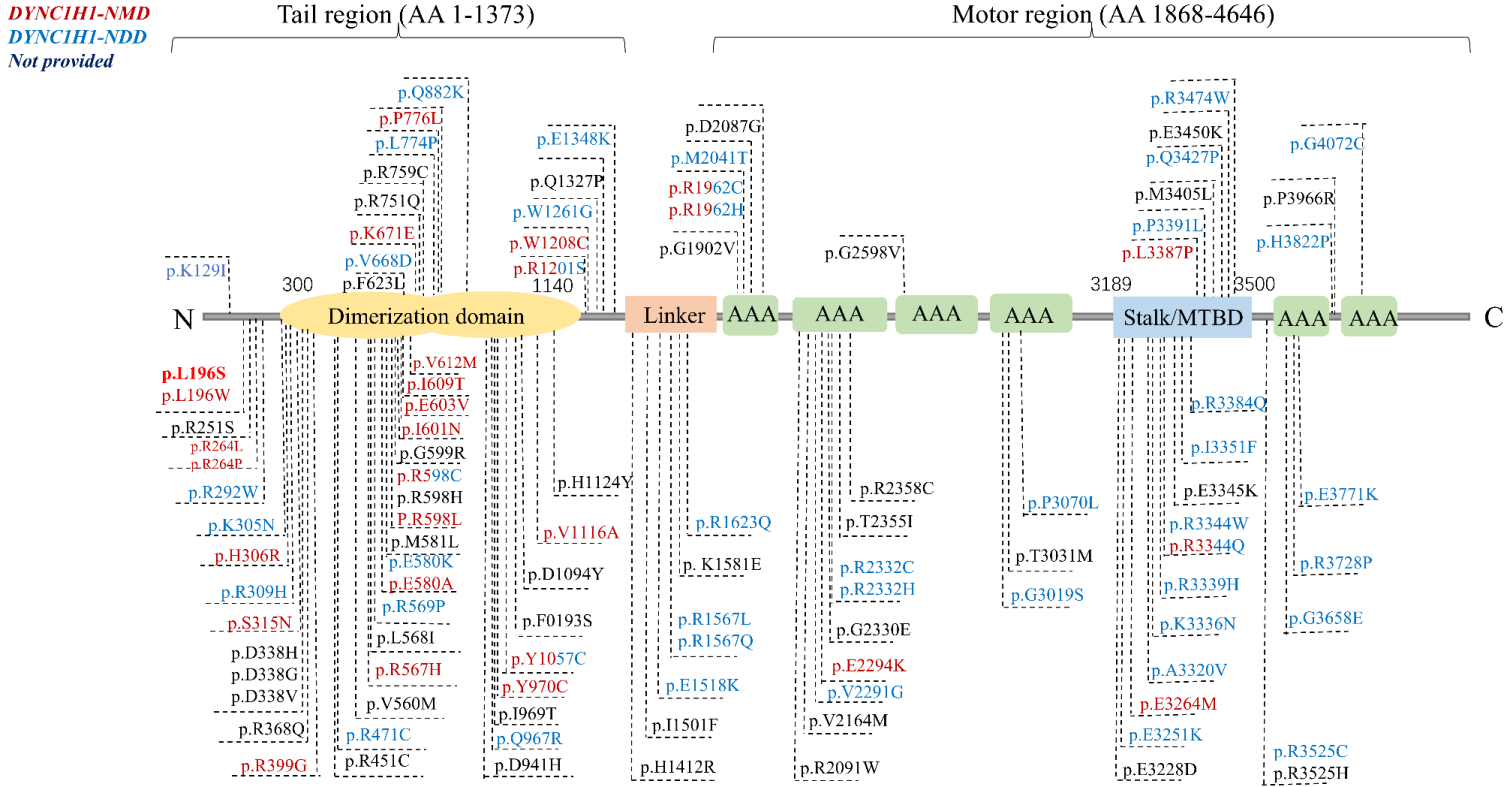
Schematic representation of *DYNC1H1* protein structure and 105 likely pathogenic or pathogenic missense variants in the ClinVar database. *DYNC1H1*-NMD variants (dark red) are mainly located in the tail region, predominantly within the dimerisation domain, whereas *DYNC1H1*-NDD variants (blue) are primarily distributed in the motor region. Four variants (p.R598C, p.Y1057C, p.R1201S, and p.R1962H) are shown in both colours because their phenotypes overlap. Variants that are not associated with definitive phenotypes were shown in black. The novel variant (p.L196S) identified in this study is shown in red bold type. Abbreviations: *DYNC1H1*-NMD, *DYNC1H1* neuromuscular disorder; *DYNC1H1-NDD*, *DYNC1H1* neurodevelopmental disorder; *MTBD*, microtubule-binding domain

The pathogenic gene of SMALED1 is *DYNC1H1*, which is located on chromosome 14q32 with 78 exons and encodes the heavy chain of cytoplasmic dynein 1. DYNC1H1 protein is divided into the tail and motor domains separated by a linker and stalk or microtubule-binding domain [10, 11] (Fig. 5). Dynein generates force through its 2 motor domains, each of which comprises a ring (15 nm in diameter) of 6 AAA^+^ modules (AAA1–AAA6) [18]. The tail domain is involved in the dimerisation of the DHCs and contacts the AAA^+^ ring through a coiled-coil stalk [19, 20]. Diseases caused by *DYNC1H1* mutation are heterogeneous, affecting the central or peripheral nervous system or both. Multiple hereditary neuromuscular *DYNC1H1*-related disorders including MCD, CMT2O, MRD13, and SMALED1 have overlapping phenotypes and share some mutation loci, making them clinically indistinguishable. Additionally, *DYNC1H1* may be related to amyotrophic lateral sclerosis (ALS). One of the pathogenic genes of ALS is *DCTN1* encoding dynactin subunit 1, which plays a key role in dynein-mediated retrograde transport of vesicles and organelles along microtubules by recruiting and tethering dynein to microtubules [21]. The legs at odd angles (*Loa*) mutation in cytoplasmic dynein has been shown to enhance mitochondrial function in an SOD1 (G93A) mouse model [22, 23]. The *DHTKD1* gene causes not only ALS, but also CMT2 and SMA [24], and ALS-frontotemporal dementia may be associated with *DYNC1H1* mutation [25]. These studies suggest a potential association between *DYNC1H1* and ALS, which will prompt scientists to further investigate the involvement of the *DYNC1H1* gene in ALS. Given the confusion and misdiagnosis engendered by current disease categories, Becker et al. [10] has recommended adopting a broader classification of (1) *DYNC1H1* neuromuscular disorders (*DYNC1H1*-NMDs) involving only the peripheral nervous system and (2) *DYNC1H1* neurodevelopmental disorders (*DYNC1H1*-NDDs), which is associated with central nervous system phenotypes. Genotype–phenotype analyses of *DYNC1H1*-related disorders have revealed significant variability in the location of *DYNC1H1* mutations [10]. Variants associated with *DYNC1H1*-NMD tend to be located in the tail region, predominantly within the dimerisation domain and mainly affect the lower limbs (similar to SMALED and CMT2O) [4], presenting as muscle weakness, foot deformities, and delayed motor development. *DYNC1H1*-NDD variants mostly cluster in the motor regions and cause varying degrees of intellectual disability including learning and speech impairment. According to this classification, an alternative diagnosis in our study is *DYNC1H1*-NMD, as our patients showed an exclusively neuromuscular phenotype and the mutation (p.Leu196Ser) was present in the tail region.

The binding of dynein complexes to dynactin and a cargo adaptor activates the processive mobility of dynein, which increases the velocity of movement [26]. Bicaudal D2 (BICD2), the predominant activating cargo adaptor [27], plays an important role in dynein-based cargo transport in neurons [28]. The N-terminal coiled-coil domain (BICD2N) of this protein binds to dynein and dynactin, and the C-terminal coiled-coil domain (BICD2C) binds to cargo [27]. In *Loa*^+/−^ mice, the *Loa* mutation (F580Y) is located in the tail domain of DHC [29]; the mice exhibit lower motor neuron degeneration, severe loss of sensory neurons, and a decreased rate of retrograde axonal transport [29–31]. It was reported that *Loa* mutation decreased dynein run-length, which impaired in vivo transport; moreover, the study provided direct evidence for communication between the dynein motor and tail domains [4]. A mutation (nudAF208V) in the tail of DYNC1H1 in the filamentous fungus *Aspergillus nidulans* (in a residue equivalent to position 186 of the human protein) decreased the frequency and speed of minus-end–directed cargo transport in vivo [32]. A number of mutations in the tail domain (e.g., K129I) that affect processive movements of dynein–dynactin cargo adaptor complexes have been identified [33], demonstrating the communication between the tail and motor domains. The mutation identified in our study (L196S) was in the tail domain and could similarly impair the function of DYNC1H1. Additionally, as a different amino acid substitution at the same nucleotide position (c.587T > G, p.L196W, rs1595597572) has been reported to be likely pathogenic, we predicted the three-dimensional structure of DYNC1H1 using AlphaFold software to assist us in determining whether L196S is pathogenic **(**Fig. 4c). The substitution of leucine with tryptophan did not appear to alter the protein structure; however, the hydrogen bond between Leu196 and Gly192 disappeared. Hydrophobic interactions and hydrogen bonding play important roles in maintaining protein stability [34]. Leucine is a hydrophobic amino acid whereas serine is hydrophilic; therefore, the L196W mutation may alter hydrophobic interactions and consequently, the stability of DYNC1H1, which could also be reduced by the loss of the hydrogen bond with Gly192. A nudAF208I mutation at the equivalent position does not cause the same defects, suggesting that even a subtle difference in the size or hydrophobicity of the amino acid side chain at this position has major consequences [32]. As of October 2022, 105 *DYNC1H1* missense mutations have been listed as likely pathogenic or pathogenic in the ClinVar mutation database (Fig. 5).

As immobilisation can directly interfere with the normal process of bone formation and accelerate the loss of bone minerals, children with motor disabilities and muscle atrophy are prone to low bone mass and fragility fractures [35]. Many studies have investigated the relationship between bone metabolism, BMD, and fracture incidence in children with SMA [35–38], and have shown that low BMD is common in this population. Long bone fractures and vertebral compression have been frequently reported. Vitamin D deficiency and up-regulation of bone resorption markers suggest that increased bone fragility and disuse osteoporosis in patients with SMA cannot be completely explained by a reduction in mechanical load on developing bone. Although the 8-year-old proband in our study suffered from chronic lower limb muscle atrophy and disability, he had not experienced any fractures and his bone metabolism markers and BMD were all within normal ranges. Likewise, his mother and 2 uncles had not experienced any fractures. To date, no other study has reported bone metabolism and BMD in patients with SMALED1, which may differ from SMA. A larger sample size is needed to investigate the detailed characteristics of bone metabolism, BMD, and fractures in children with SMALED1.

There were some limitations to this study. First, histopathology and subcellular structure were not investigated. Second, we did not perform in vitro and in vivo experiments to elucidate the pathogenic mechanism by which the identified mutation causes the disease phenotype. Third, as the sample size was small, the skeletal characteristics of the SMALED1 patients could not be further analysed.

## Conclusion

In the present study, a novel *DYNC1H1* mutation (c.587T > C, p.Leu196Ser) was identified as the cause of SMALED1 in a Chinese family. Our study broadens the mutational spectrum of *DYNC1H1* and can facilitate genetic counselling and prenatal diagnosis. The phenotype overlap with dHMN can complicate clinical diagnosis of SMALED1; therefore, WES combined with Sanger sequencing is recommended to avoid delaying diagnosis. Bone metabolism markers and BMD of the proband in our study were all normal, but further research on bone metabolism and BMD in patients with SMALED1 is needed in the future.

## Data Availability

The datasets generated in the current study are available in the Mendeley repository at https://data.mendeley.com/datasets/t5p499pmkv/1.
